# Thermodynamic and Thermal Analyze of *N*,*N*-Dimethylformamide + 1-Butanol Mixture Properties Based on Density, Sound Velocity and Heat Capacity Data

**DOI:** 10.3390/molecules28124698

**Published:** 2023-06-11

**Authors:** Magdalena Tyczyńska, Aleksandra Dentkiewicz, Małgorzata Jóźwiak

**Affiliations:** Department of Physical Chemistry, Faculty of Chemistry, University of Lodz, Pomorska 165, 90-236 Lodz, Poland; ola.dentkiewicz98@gmail.com (A.D.); malgorzata.jozwiak@chemia.uni.lodz.pl (M.J.)

**Keywords:** *N*,*N*-dimethylformamid + 1-butanol mixture, density, sound velocity, molar heat capacity, excess functions

## Abstract

The present paper contains data on the density (ρ), sound velocity (u), and specific heat capacity $\left(c_{p}\right)$ of the mixture of *N*,*N*-dimethylformamide + 1-butanol (DMF + BuOH) determined in the entire concentration range of solution and in the temperature range (293.15–318.15) K. The analysis of thermodynamic functions such as isobaric molar expansion, isentropic and isothermal molar compression, isobaric and isochoric molar heat capacity, as well as their excess functions $\left(E_{p,m}^{E},K_{S,m}^{E},K_{T,m}^{E},C_{p,\;m}^{E},C_{V,\;m}^{E}\right)$ and also $V_{m}^{E}$ was undertaken. The analysis of changes in the physicochemical quantities was based on consideration of the system in terms of intermolecular interactions and resulting changes in the mixture structure. The results available in the literature were confusing during the analysis and became the reason for our decision to thoroughly examine the system. What is more, for a system whose components are widely used, there is very scarce information in the literature regarding the heat capacity of the tested mixture, which was also achieved and presented in this publication. The conclusions drawn from so many data points allow us to approximate and understand the changes that occur in the structure of the system due to the repeatability and consistency of the obtained results.

## 1. Introduction

Thermodynamic properties are a valuable source of information on the interaction between solute and solvent molecules. In particular, the combination of techniques such as density, sound velocity, and specific heat capacity gives a range of data allowing one to analyze the changes in the structure of mixed solvents. Regarding the thermodynamic properties, it appeared that volumes, heat capacities, expansivities, and compressibilities are sensitive to structural changes and allow prediction of the type of interactions prevailing in the liquid mixtures. The density, ultrasonic, and thermodynamic studies with the change of one component of the solution are of high value and practical importance in industry. Such data are also used in manufacturing process control and other significant fields.

Alcohol-containing mixtures are gaining in popularity. Such solutions are used in the pharmaceutical and cosmetic industries, in high-energy battery technologies, or in organic synthesis [1,2]. Great interest in the product requires a better understanding of the influence of the liquid structure on its macroscopic properties, including density, speed of ultrasound propagation, and heat capacity [3,4]. 1-Butanol (BuOH) is used as a perfume ingredient, a solvent for the extraction of essential oils, and an extractant in the production of antibiotics, hormones, and vitamins [5]. It is also present in the cosmetics industry, mainly in make-up removers, nail care, and shaving products [5]. BuOH is also used in the food industry as a flavoring agent in cream and baked goods [5]. *N*,*N*-dimethylformamide (DMF) has many useful properties, making it an exceptionally good cosolvent [6]. Secondary and tertiary amides play a role in constructing the backbone conformation of peptides and proteins [7]. DMF is used in the formation of pesticides and in the manufacture of synthetic leathers, fibers, films, and surface coatings [8,9]. It is also used in the production of vinyl and acrylic polymers. It is an extractant as well as an absorbent of gases [10]. The mixture of DMF with supercritical CO_2_ is of great interest in the extraction of supercritical fluids in separation processes [11,12,13]. It is well known that amides interact with alcohols through hydrogen bonding, and the addition of DMF to alcohol causes characteristic changes in mixture structure. Due to this fact, it is interesting to know the properties of the DMF and 1-buthanol mixture. The available data on this mixture are often focused mainly on densimetric data, sometimes enriched with ultrasound or viscosity techniques [4,14,15,16,17,18,19,20,21].

However, the results obtained by the researchers are not entirely consistent and often cover a narrow temperature range, as well as a few points that determine the change in the composition of the mixture [4,14,15,16,17,18,19,20,21]. The heat capacity data in the available literature is very scarce [22], and there is no complete information on the value of the specific heat capacity in the entire range of the composition of the mixture and the wide temperature changes. The data obtained in this work include analysis of selected physicochemical quantities, e.g., excess molar volume $\left(V_{m}^{E}\right)$, also excess partial molar volume of 1-butanol ${(V}_{m,BuOH}^{E})$ and *N*,*N*-dimethylformamide ${(V}_{m,DMF}^{E})$, molar volume expansion $\left(E_{p,m}\right)$, molar isentropic ($K_{S,m}$) and isothermal ($K_{T,m}$) compressibility, isobaric $\left(C_{p,m}\right)$ and isochoric $\left(C_{V,m}\right)$ molar heat capacity as well as their excess values $(E_{p,m}^{E},K_{S,m}^{E},K_{T,m}^{E},$$C_{p,m}^{E},C_{V,\;m}^{E})$ of the DMF + BuOH system at six temperatures in the whole concentration range. The data are available in the literature [4,14,15,16,17,18,19,20,21]. The tested system so far does not include small and precise changes in the concentration of the mixture and such a wide range of temperatures as presented in our work. The change in concentration is 0.05 mole fraction, which allowed us to show characteristic changes in analyzed functions not previously listed. Connecting so many data points gives the opportunity to draw a variety of valuable conclusions regarding changes in the structure of the system [23,24]. Moreover, we determined the thermal properties of the system by making attempts to measure the isobaric specific heat capacity $\left(c_{p}\right)$. In the literature, there are no data presenting isobaric $\left(C_{p,m}\right)$ and isochoric $\left(C_{V,m}\right)$ molar heat capacities of the system. Such data, in combination with density and ultrasound velocity, in a temperature range of 293.15–318.15 K, is worth attention due to its coherent results.

## 2. Results and Discussion

### 2.1. Volumetric Properties

The results obtained from the density tests of the DMF + BuOH mixture (Table S1 supplementary materials) show that the values increase with increasing DMF content in the mixture and decrease systematically with increasing temperature over the whole range of mixture compositions. To study the properties of real solutions, excess functions are used, which determine the difference between the magnitude of a given molar thermodynamic function in a real solution and its magnitude in an ideal solution. The excess properties were calculated using the following expression:

(1) $$Z^{E}=Z-Z^{\mathrm{id}}$$

where $Z^{E}$ is the excess quantity of the property *Z* and $Z^{id}$ is the corresponding ideal value [25].

In this paper, we will present and analyze six excess functions $(V_{m}^{E},\;E_{p,m}^{E},\;K_{S,m}^{E},\;K_{T,m}^{E},\;$$C_{p,m}^{E},\;C_{V,m}^{E})$ calculated according to Equation (1). In order to calculate excess molar volume $\left(V_{m}^{E}\right)$, the molar volume of the mixture was obtained according to Equation (2):

(2) $$V_{m}=\frac{x_{1}M_{1}+x_{2}M_{2}}{\rho }$$

where: ρ is the density of the DMF + BuOH mixture, $x_{1}$, $x_{2}$ and $M_{1}$, $M_{2}$ are the mole fractions and molar masses of the mixture components, respectively, i.e., BuOH (1), DMF (2).

**Figure 1:**
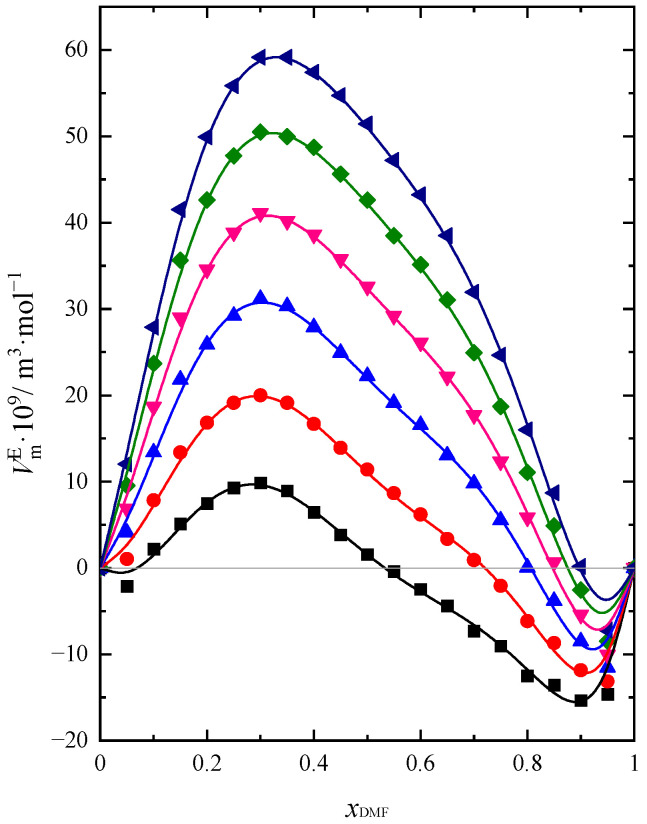
Excess molar volume ($V_{m}^{E}$) of DMF + BuOH mixture: ■ *T* = 293.15 K, ● *T* = 298.15 K, ▲ *T* = 303.15 K, ▼ *T* = 308.15 K, ◆ *T* = 313.15 K, ◀ *T* = 318.15 K. Solid lines are obtained using Redlich-Kister Equation (4).

In order to determine the changes taking place in a real solution in relation to an ideal solution in a binary mixture, the values of excess molar volume ($V_{m}^{E})$ have been executed. $V_{m}^{E}$ values of the mixture in the whole composition range and at the temperature range (293.15–318.15 K) were calculated according to Equation (3) and presented in Figure 1:

(3) $$V_{m}^{E}=V_{m}-V_{m}^{\mathrm{id}}=V_{m}-(x_{1}V_{1}^{*}+x_{2}V_{2}^{*})$$

where: $V_{m}$ is the molar volume of the (DMF + BuOH) mixture, $V_{m}^{id}$ is the volume of the ideal mixture, $V_{1}^{*}$, $V_{2}^{*}$ are the molar volumes of pure compounds, i.e., BuOH (1), DMF (2).

The values of excess functions and, among others, the $V_{m}^{E}$ of the DMF + BuOH mixture were fitted to the polynomial of Redlich−Kister type:

(4) $$V_{m}^{E}=x_{1}x_{2}\sum_{j=0}^{n}A_{j}{\left(1-2x_{1}\right)}^{j}$$

(5) $$\frac{V_{m}^{E}}{x_{1}x_{2}}=\sum_{j=0}^{n}A_{j}{\left(1-2x_{1}\right)}^{j}$$

where $A_{j}$ is the polynomial coefficient calculated by the least-squares method using Equation (5).

As can be seen in Figure 1, the excess molar volume exhibits positive values in a solution with a predominantly BuOH content in the mixture. $V_{m}^{E}$ increases when small amounts of DMF are added to pure BuOH and passes through a maximum, which appears depending on the temperature between $x_{DMF}$ ≈ 0.3 and $x_{DMF}$ ≈ 0.35 values. Above this value of mole fraction, $V_{m}^{E}$ values are decreasing and reach the minimum value at $x_{DMF}$ ≈ 0.9. It is noteworthy that negative values for $V_{m}^{E}$ are obtained for different mixture compositions depending on the temperature. The mole fraction of DMF, in which we observe the change of sign of the $V_{m}^{E}$ function, increases with increasing temperature from the value $x_{DMF}$ ≈ 0.5 for 293.15 K to the value $x_{DMF}$ ≈ 0.9 for 318.15 K. One can notice that the volume contraction of the DMF + BuOH mixture increases when the molar fraction of DMF increases above 0.5 and decreases with increasing temperature. In the literature, you can find several reports in which an attempt was made to determine the value of $V_{m}^{E}$ for the DMF + BuOH mixture [14,15,16,19,20,21]. However, the research results presented in these articles are divergent. Similar results to ours were obtained by Rao and Reddy [14] at 303 K and Garcia et al. [21], but only at 298 K. In the other works, the results diverged from each other and from ours. Moreover, some authors obtained negative values of $V_{m}^{E}$ in the whole range of compositions [15,19], which was completely inconsistent with the results obtained by other researchers, including ours. Such ambiguous data and conclusions prompted us to study the properties of this mixture with more accuracy using the three different test methods mentioned earlier.

The sign and magnitude of the excess functions may be attributed to the result of an appropriate combination of the following three major effects. The mutual dissociation of the components due to the addition of the second component, the formation of hydrogen bonds between different molecules, steric hindrance, as well as the geometry of molecular structure, could be the reasons for the resistance of the molecules. For DMF + PrOH, according to the results obtained by us in our previous work [26], the values were negative over the entire range of the mixture composition. Excess molar volumes are more negative in systems with lower alcohols, which may be attributed to strong interactions between different molecules and different molecular sizes [27]. Such properties cause volume contraction in these mixtures. For the DMF + BuOH system, the small increase in the size of the alcohol molecule (extra –CH_2_ group in 1-butanol compared to DMF + PrOH [26]) gives completely different results and appears in volume expansion when DMF is added to BuOH. These values are positive in the BuOH-rich region. Mostly for the DMF + BuOH system, the results obtained in their absolute value are 1.5 to 2 times smaller than for the DMF + PrOH mixture; however, in some compositions, they are several or ten times smaller than for the DMF + PrOH mixture.

Alcohols are strongly hydrogen-bonded in their pure state. Their molecules are self- and cross-associated [28,29]. The degree of association decreases with increasing alcohol chain length. Thus, the addition of DMF to pure BuOH breaks the hydrogen bonds between molecules in the structure of the alcohol network, which produces a positive contribution to $V_{m}^{E}$. The results obtained on the basis of the dielectric study prove that in a solution with a predominant content of BuOH, the largest changes in the structure of hydrogen bonds occur in the DMF + BuOH solution [30]. At the same time, an increase in the length of the alcohol chain causes a steric hindrance that also contributes to the increase in the real volume of the DMF + BuOH mixture. There is also not much difference in the molecular size of these two compounds, which also makes mutual accommodation difficult. The presence of hydrogen bonds between the components of the mixture tested was also confirmed by other researchers [4,20,30,31]. Prajapati [30], based on the analysis of parameters obtained from the dielectric study, provides confirmation of the formation of hydrogen bonds between DMF and BuOH molecules and weak dipole-dipole interactions between the components of the mixture. In contrast to the DMF + PrOH mixture, it is known that the interactions between different molecules are very weak in the DMF + BuOH solution [32]. Therefore, we observe an increase in $V_{m}^{E}$ with increasing DMF content in the mixture to $x_{DMF}$ ≈ 0.3. The maximum observed at this mole fraction of DMF is related to the fact that in this range of mixture composition there are probably the weakest interactions between the components of the mixture, which also contributes positively to the $V_{m}^{E}$ [32]. Despite the fact that there is probably still the possibility of forming some hydrogen bonds between DMF and BuOH molecules, the excess BuOH amount is a competitive factor in the formation of hydrogen bonds between different molecules. According to the researchers [32], the binding energy –O–H···O=C decreases when another alcohol molecule approaches with its oxygen atom favorablely oriented for “change”. As a consequence, instead of hydrogen bonding between DMF and BuOH molecules, the bonding appears between alcohol molecules rather more. When the amount of BuOH prevails in the DMF + BuOH system, there is a greater tendency to create hydrogen bonds between BuOH molecules than between different species. As a result of the specific arrangement of neighboring molecules around the DMF and BuOH molecules that form the bond, this interaction is weakened, which is confirmed by the negative $\varepsilon_{0}^{E}$ values in the entire concentration range of the solution obtained by Prajapati [30]. This parameter assumes the largest negative deviation for a mixture with the composition $x_{DMF}$ ≈ 0.3; hence, we observe a maximum on the dependency $V_{m}^{E}$ = *f*($x_{DMF})$. The dissociation of bonding between pure components presumably became the main reason for the volume expansion of the tested system. When $x_{DMF}$ > 0.3 $V_{m}^{E}$ values decrease and become negative when $x_{DMF}$ > 0.5 (293.15 K). In a solution with a predominant DMF content in the mixture, they can observe small volume contractions. The volume contraction value is similar to that observed for the DMF + PrOH system in the same concentration area of the mixture. When DMF starts to prevail in the solution, dipole–dipole interactions are most likely to begin to prevail in DMF, leading to the creation of hydrogen bonding between DMF and BuOH molecules.

The analysis of the excess partial molar volume of both components of the mixture may help to explain the observed changes in this range of the mixture composition. The volume of the solution, in the case of a binary mixture, is the sum of the partial molar volumes of both components:

(6) $$V=n_{BuOH}\cdot V_{m,BuOH}+n_{DMF}\cdot V_{m,DMF}$$

where: $n_{BuOH}$, $n_{DMF}$—mole fraction of BuOH i DMF, respectively; $V_{m,BuOH}$; $V_{m,DMF}$—partial molar volume of BuOH and DMF, respectively. $V_{m,BuOH}$ and $V_{m,DMF}$ we can calculate using the following Equations (7) and (8):

(7) $$V_{m,DMF}=V_{m}+x_{BuOH}\left(\frac{\partial V_{m}}{\partial x_{DMF}}\right)$$

(8) $$V_{m,BuOH}=V_{m}+x_{DMF}\left(\frac{\partial V_{m}}{\partial x_{BuOH}}\right)$$

where: $V_{m}$—molar volume of the real solution (DMF + BuOH), $x_{DMF}$, $x_{BuOH}$—mole fraction of DMF and BuOH.

An analysis of changes in the partial molar volume of the components of a mixture can be represented by the excess partial molar volume ($V_{DMF}^{E}$, $V_{BuOH}^{E}$). These values can be calculated using Equations (9) and (10):

(9) $$V_{m,DMF}^{E}=V_{m,DMF}-V_{DMF}^{*}$$

(10) $$V_{m,BuOH}^{E}=V_{m,BuOH}-V_{BuOH}^{*}$$

where: $V_{m,DMF}^{E}$, $V_{m,BuOH}^{E}$—excess partial molar volume of DMF and BuOH, respectively; $V_{DMF}^{*}$, $V_{BuOH}^{*}$—molar volume of pure DMF and BuOH.

**Figure 2:**
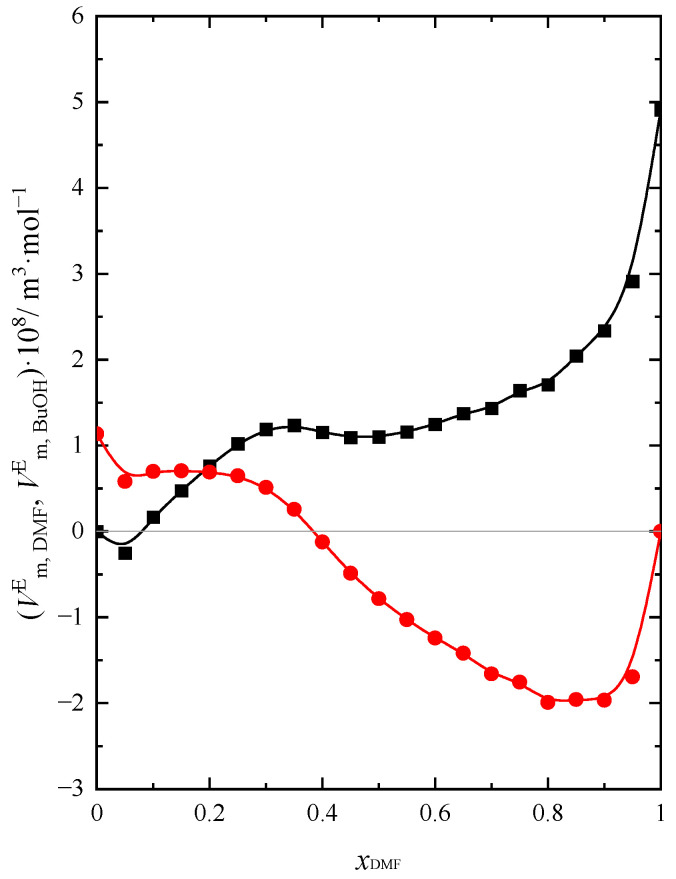
Excess partial molar volume ($V_{m,DMF}^{E}$, $V_{m,BuOH}^{E}$) of: ● DMF, ■ BuOH in the DMF + BuOH mixture at *T* = 293.15 K.

The results for partial and excess partial molar volumes of DMF and BuOH, $V_{m,DMF}^{E}$ and $V_{m,BuOH}^{E}$, are presented in Tables S4–S7 in the supplementary materials and presented in Figure 2 at *T* = 293.15 K.

The values of $V_{m,DMF}^{E}$ and $V_{m,BuOH}^{E}$ express the difference between the value of the partial molar volume of DMF or BuOH in the solution and the molar volume of each of the components in their pure form. Based on the analysis of Figure 2, one can notice that for DMF, the values of $V_{m,DMF}^{E}$ are negative when $x_{DMF}$ > 0.4 at 293.15 K. This means that DMF contributes negatively to the real volume of the mixture in this composition range of the solution. Although $V_{m,BuOH}^{E}$ assumes positive values in the same concentration range, the amount of DMF prevails over the amount of BuOH when $x_{DMF}$ > 0.4. Having been aware that DMF is a weakly associated liquid in contrast to BuOH [30], the results obtained allow us to conclude that the negative contribution to the real volume of the mixture is made by the DMF molecules ($V_{m,DMF}^{E}<0).$ This is presumably one of the main factors causing the slight contraction of the volume of the mixture, causing us to observe $V_{m}^{E}$ < 0 when $x_{DMF}$ > 0.5. Analysis of data on the partial molar volume of each component in the DMF + BuOH mixture (Tables S4 and S5 in the supplementary materials) allows one to observe that the dependencies $V_{m,DMF}=f(x_{DMF})$ and $V_{m,BuOH}=f(x_{DMF})$ show only a little change with increasing DMF content in solution. This proves that chemical entities, such as various types of associations or complexes built of both molecules of the solution components, are most likely not formed in the system. Otherwise, we would observe characteristic changes in the course of both functions, along with an increase in the content of one of the components of the mixture, as in the case of an aqueous solution of *N*,*N*-dimethylformamide [33], which also confirms the conclusions drawn earlier. The partial molar volume of DMF decreases slightly as the DMF content of the mixture increases at a higher temperature. Garcia et al. [19] presented a similar course of this dependence at a temperature of 298.15 K. In addition, $V_{m,DMF}$ takes the highest value at the highest temperature. As determined by Zegers and Somsen [22], the value of $V_{m,DMF}$ in pure BuOH is equal to 7.779 × 10^−5^ m^3^·mol^−1^ and is close to the one obtained by us (7.749 × 10^−5^ m^3^·mol^−1^). The course of the dependency $V_{m,BuOH}=f(x_{DMF})$ is opposite to that observed for DMF. $V_{m,BuOH}$ values increase slightly with increasing DMF content. The influence of temperature on the values of $V_{m,BuOH}$ is analogous to that of $V_{m,DMF}$. Zegers and Somsen [22] also determined $V_{m,BuOH}$ in pure DMF, which is equal to 9.197 × 10^−5^ m^3^·mol^−1^. The value obtained by them is close to the one determined by us (9.208 × 10^−5^ m^3^·mol^−1^).

Using the density values of the mixture at six temperatures, the coefficient of thermal expansion (*α_p_*) was calculated using Equation (11):

(11) $$\alpha_{p}=\frac{1}{V_{m}}{\left(\frac{\partial V_{m}}{\partial T}\right)}_{p}=-\frac{\partial \ln\rho }{\partial T}=-\frac{\partial \rho }{\rho \partial T}$$

$V_{m}$ was calculated with Equation (12) [34]:

(12) $$V_{m}=a_{2}{\left(T-273.15\right)}^{2}+a_{1}(T-273.15)+a_{0}$$

The values of molar isobaric expansion ($E_{p,m}$) were calculated using Equation (13) [25]:

(13) $$E_{p,m}^{}=\alpha_{p}\cdot V_{m}$$

**Figure 3:**
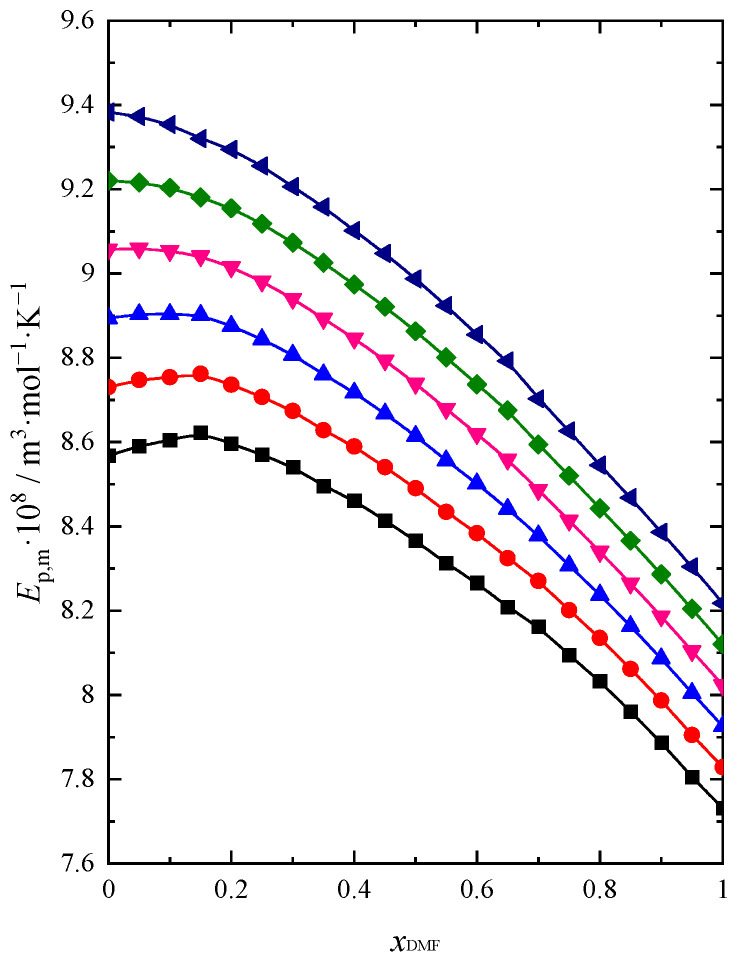
Isobaric molar expansion ($E_{p,m}$) of DMF + BuOH mixture: ■ *T* = 293.15 K, ● *T* = 298.15 K, ▲ *T* = 303.15 K, ▼ *T* = 308.15 K, ◆ *T* = 313.15 K, ◀ *T* = 318.15 K.

The obtained results of $E_{p,m}$ as a function of the DMF molar fraction are presented in Figure 3.

The isobaric molar expansion reaches its highest value for pure BuOH and decreases with increasing DMF in the mixture. The $E_{p,m}$ values increase with increasing temperature over the whole composition range of the mixture, which seems logical due to the increase in thermal movements at higher temperatures. Such a behavior of the system in the BuOH-rich region is most likely related to the breaking of hydrogen bonds in BuOH after adding DMF to the solution. This causes greater changes in volume expansion with an increase in temperature. With a high BuOH content in the mixture, a slight maximum is visible at the two lowest temperatures (293.15 K, 298.15 K). For higher temperatures on the $E_{p,m}=f\left(x_{DMF}\right)$ dependency, we observe only a change in the slope of the function. A greater effect of temperature on the value of this function is observed when BuOH prevails in the mixture. As the DMF content increases, the temperature differentiates the $E_{p,m}$ values of the mixture less. Small changes in the value of the isobaric molar expansion in the DMF-rich region show that the structure of this solvent remains only slightly associated with dipole–dipole interactions.

**Figure 4:**
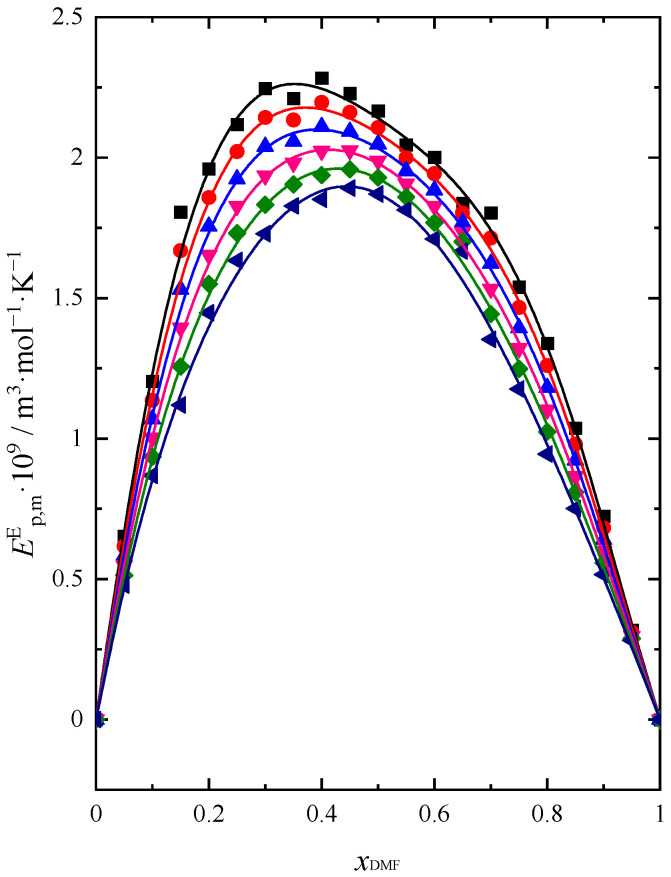
Excess molar isobaric expansion ($E_{p,m}^{E}$) of (DMF + BuOH) mixture: ■ *T* = 293.15 K, ● *T* = 298.15 K, ▲ *T* = 303.15 K, ▼ *T* = 308.15 K, ◆ *T* = 313.15 K, ◀ *T* = 318.15 K. Solid lines are obtained using Redlich-Kister Equation (4).

Using the calculated values of $E_{p,m}$ and Equations (14) and (15) [25], excess molar isobaric expansion was determined ($E_{p,m}^{E}$) and presented in Figure 4:

(14) $$E_{p,m}^{id}=\alpha_{p}^{id}\cdot V_{m}^{id}=\left(\varphi_{BuOH}\cdot \alpha_{p,BuOH}^{*}+\varphi_{DMF}\cdot \alpha_{p,DMF}^{*}\right){\cdot (x}_{BuOH}\cdot V_{BuOH}^{*}+x_{DMF}\cdot V_{DMF}^{*})$$

(15) $$E_{p,m}^{E}=E_{p,m}-E_{p,m}^{id}$$

where: $\varphi_{BuOH},\;\varphi_{DMF}$ are volume fractions of BuOH and DMF and $\alpha_{p,BuOH}^{*},\;\alpha_{p,DMF}^{*}$ are the coefficients of thermal expansion of pure BuOH and DMF, respectively.

Excess isobaric molar expansion in the entire concentration range has positive values. A real solution has a greater ability to thermally expand relative to an ideal solution. In the range of 0.4 ≤ $x_{DMF}$ ≥ 0.5 (depending on the measurement temperature), a maximum appears, which proves the occurrence of characteristic changes in the interactions between molecules in this composition range. The value of $E_{p,m}^{E}$ is the lowest at *T* = 318.15 K, which means that at the highest temperature, the volumetric expansion of a real solution is the smallest and increases with decreasing temperature. It should be expected that at the lowest temperatures, the interactions between molecules will be stronger, with the intermolecular interactions weakening with increasing temperature.

### 2.2. Sound Velocity and Heat Capacity

Based on density and sound velocity measurements (Tables S1 and S2 in the supplementary materials), the isentropic compressibility coefficient $\left(\kappa_{S}\right)$ and molar isentropic compression $\left(K_{S,m}\right)$ were calculated according to Equations (16) and (17) in the whole temperature range:

(16) $$\kappa_{S}=-\frac{1}{V_{m}}{\left(\frac{\partial V_{m}}{\partial p}\right)}_{S}=\frac{1}{u^{2}\rho }$$

(17) $$K_{S,m}=V_{m}\cdot \kappa_{S}$$

where $\kappa_{S}$ is the isentropic compressibility coefficient, $K_{S,m}$ is the molar isentropic compression*,* u is the sound velocity of the DMF + BuOH mixture, ρ is the experimental value of the solution’s density.

Based on the obtained data, such as the isentropic compressibility coefficient $\left(\kappa_{S}\right)$, the coefficient of isobaric thermal expansibility ($\alpha_{p})$ and experimentally gained data on density (ρ) and specific heat capacity ($c_{p})$ of the tested DMF + BuOH solution, which are presented in Tables S1 and S3 in the supplementary materials, the values of isothermal compressibility efficient $\left(\kappa_{T}\right)$ and isothermal molar compression $\left(K_{T,m}\right)$ were calculated using Equations (18) and (19):

(18) $$\kappa_{T}=\kappa_{S}+\frac{\alpha_{p}^{2}T}{c_{p}\rho }$$

(19) $$K_{T,m}=V_{m}\cdot \kappa_{T}$$

The obtained results of $\kappa_{S}$ and $\kappa_{T}$ for the whole composition and temaprature range are presented in Table S8 (supplementary materials). The course of changes in isentropic and isothermal molar compression as a function of concentration and temperature is very analogous. $K_{S,m}$ and $K_{T,m}$ reach similar values. Both isentropic and isothermal molar compression decrease with increasing DMF content in the mixture. This is in agreement with the results of other researchers [35]. The highest value of $K_{S,m}$ and $K_{T,m}$ is observed for pure BuOH due to the hydrogen bonds existing in the alcohol structure. $K_{S,m}$ and $K_{T,m}$ increases with the concentration of alcohol. It is principally associated with an increase in compressibility due to structural changes in the mixture that lead to a decrease in ultrasonic velocity [36]. Adding DMF to BuOH breaks these bonds and creates new, weaker ones between the DMF and BuOH molecules. This leads to a closer arrangement of the molecules. In the DMF-rich region, only the dipol-dipol interaction prevails. Most likely, this phenomenon contributes to a decrease in the compressibility of the system with increasing mole fractions of DMF. The compressibility of the system increases with increasing temperature. A greater effect of temperature on the value of isentropic and isothermal molar compression is visible for solutions in which the content of BuOH prevails. When there is more DMF in the system, $K_{S,m}$ and $K_{T,m}$ depends on the temperature.

In order to better understand the nature of the interactions between the components of the mixture and the nature of molecular agitation in dissimilar molecules, excess molar isentropic compression ($K_{S,m}^{E}$) and excess molar isothermal compressibility ($K_{T,m}^{E}$) were determined. These are found to be sensitive to differences in the size and shape of molecules [37]. For this purpose $K_{S,m}^{E}$ and $K_{T,m}^{E}$ values were calculated according to Equations (20)–(24) [25]:

(20) $$K_{S,m}^{E}=K_{S,m}-K_{S,m}^{id}$$

(21) $$K_{S,m}^{id}=V_{m}^{id}\cdot \kappa_{S}^{id}$$

(22) $$\begin{array}{l} \kappa_{S}^{\mathrm{id}}=\varphi_{1}\cdot \kappa_{S,1}^{*}+\varphi_{2}\cdot \kappa_{S,2}^{*}+\\ +T\left[\frac{\varphi_{1}V_{1}^{*}\cdot {\left(\alpha_{p,1}^{*}\right)}^{2}}{C_{p,1}^{*}}+\frac{\varphi_{2}V_{2}^{*}\cdot {\left(\alpha_{p,2}^{*}\right)}^{2}}{C_{p,2}^{*}}-\frac{\left(x_{1}V_{1}^{*}+x_{2}V_{2}^{*}\right)\cdot {\left(\varphi_{1}\alpha_{p,1}^{*}+\varphi_{2}\alpha_{p,2}^{*}\right)}^{2}}{x_{1}C_{p,1}^{*}+x_{2}C_{p,2}^{*}}\right] \end{array}$$

(23) $$K_{T,m}^{E}=K_{T,m}-K_{T,m}^{id}$$

(24) $$K_{T,m}^{id}=V_{m}^{id}\cdot \kappa_{T}^{id}=(\varphi_{1}\cdot \kappa_{T,1}^{*}+\varphi_{2}\cdot \kappa_{T,2}^{*})\cdot (x_{1}\cdot V_{1}^{*}+x_{2}\cdot V_{2}^{*})$$

where: ${Κ}_{S,m}^{E},\;{Κ}_{T,m\;}^{E}$ represent excess molar isentropic and isothermal compression, ${Κ}_{S,m}$ and ${Κ}_{T,m}$ represent the molar isentropic and isothermal compression, and ${Κ}_{S,m}^{id}$ and ${Κ}_{T,m}^{id}$ their molar values for an ideal mixture; $\kappa_{S,i}^{*},\kappa_{T,i}^{*}$ the isentropic and isothermal compressibility coefficients of pure components **1** (BuOH) and **2** (DMF), $\varphi_{i}$ the volume fraction of the mixture components; $C_{p,i}^{*}$ represents the isobaric molar heat capacity of pure BuOH (1) and DMF (2) calculated on the basis of the obtained $c_{p}$ values (Table S3 in the supplementary materials).

**Figure 5:**
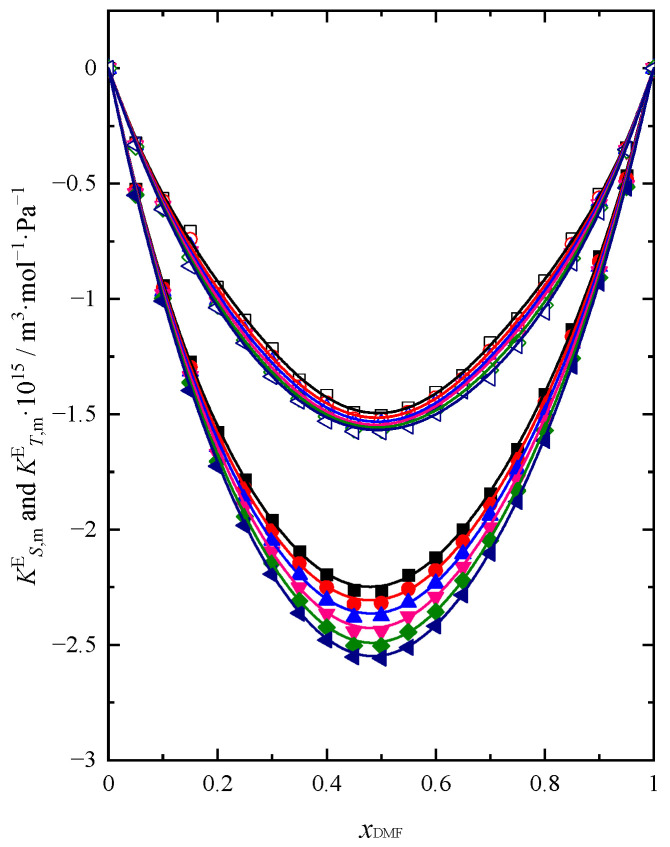
Excess molar isentropic ($K_{S,m}^{E})$ (full symbol) and isothermal $\left(K_{T,m}^{E}\right)$ (open symbol) compression of DMF + BuOH mixture at temperature: ■ *T* = 293.15 K, ● *T* = 298.15 K, ▲ *T* = 303.15 K, ▼ *T* = 308.15 K, ◆ *T* = 313.15 K, ◀ *T* = 318.15 K. Solid lines are obtained using Redlich-Kister Equation (4).

The courses of both functions $K_{S,m}^{E}=f(x_{DMF})$ and $K_{T,m}^{E}=f\left(x_{DMF}\right)$ in the whole temperature range are shown in Figure 5.

The excess molar isentropic and isothermal compressions have negative values. The same trend can be seen in studies published by Thirumaran et al. [4], Rao and Reddy [14], and Acree [35]. This parameter takes negative values, and the minimum is observed at $x_{DMF}$ ≈ 0.45. It can be concluded that the real solution is less compressible than the ideal solution. At $x_{DMF}$ ≈ 0.45, where there is a minimum on the function curves $K_{S,m}^{E}=f(x_{DMF})$ and $K_{T,m}^{E}=f\left(x_{DMF}\right)$ visible, apparently there are characteristic changes in the interactions between the particles of the real mixture in relation to the ideal solution, causing more and more difficulties in the compression of the system. With the addition of DMF to the pure BuOH, the excess compressibility rapidly decreases up to $x_{DMF}$ ≈ 0.45, caused by the rupture of hydrogen bonds in the pure BuOH occurring during mixing. In this range of composition, we also observe probably the weakest interactions between the components of the mixture [30,32]. The lower compression in the solution is observed for systems with lower alcohols, which can be attributed to strong interactions between different molecules and different molecular sizes causing stronger mutual accommodation of components [26,27]. Such a situation was observed for the DMF + PrOH mixture [26]. Since the size of both BuOH and DMF molecules is similar, the weakening and breaking of bonds and the lack of interstitial accommodation due to similar molecular sizes may be the reasons for decreasing the compressibility of the DMF + BuOH system in the BuOH-rich region. In the DMF-rich region, with the increasing importance of interactions between different molecules and dipole–dipole interactions in DMF, we can observe the decreasing negative values for $K_{S,m}^{E}$ and $K_{T,m}^{E}$. The value of $K_{S,m}^{E}$ decreases with increasing temperature. Thus, the lower the temperature, the greater the compressibility of a real solution relative to that of an ideal solution. At *T* = 293.15 K, the real system will show the least negative $K_{S,m}^{E}$ and $K_{T,m}^{E}$ of the mixture compared to the other temperatures.

**Figure 6:**
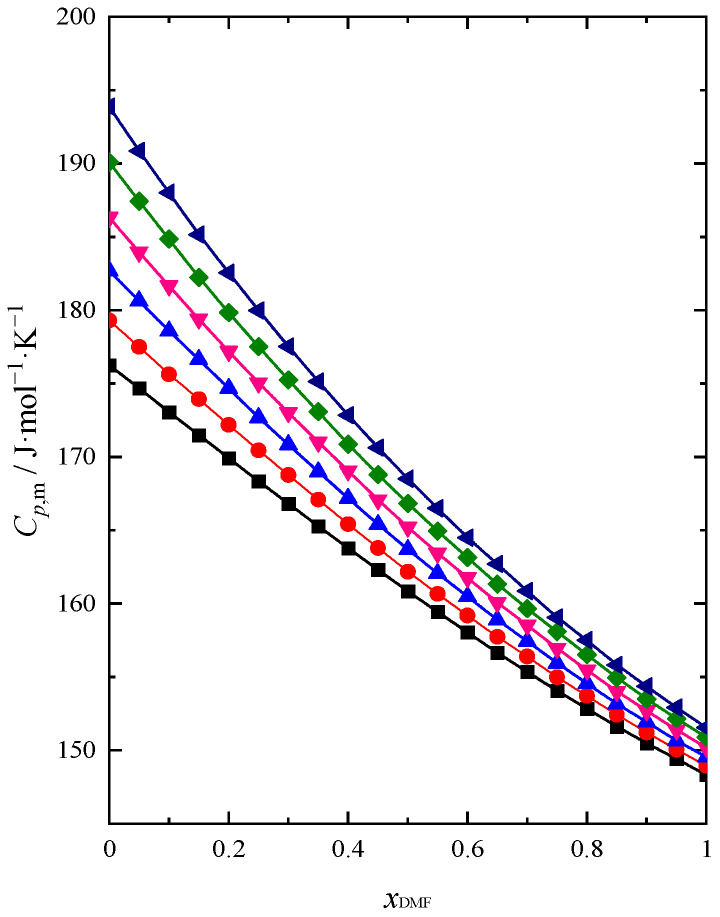
Isobaric molar heat capacity ($C_{p,m}$) of (DMF + BuOH) mixture: ■ *T* = 293.15 K, ● *T* = 298.15 K, ▲ *T* = 303.15 K, ▼ *T* = 308.15 K, ◆ *T* = 313.15 K, ◀ *T* = 318.15 K.

The values of isobaric molar heat capacity ($C_{p,m}$) were calculated using the specific heat capacity $\left(c_{p}\right)$ obtained from the experiment (Table S3 in the supplementary materials). The results for the whole composition range of the mixture and for six temperatures (293.15–318.15 K) were calculated and presented in Figure 6 and in Table S9 (supplementary materials).

Heat capacity and thermal analysis data for the DMF + BuOH systems have not been published earlier. As can be seen from Figure 6, the $C_{p,m}$ values decrease with increasing DMF content. With increasing temperature $C_{p,m}$ values are increasing. A greater temperature differentiation is also visible in the BuOH-rich region compared to the area of DMF that prevails. An analogous course of dependence with an increase in DMF content for this system (DMF + BuOH) and changes under the influence of temperature was visible in the course of the previously discussed functions ${Κ}_{S,m},\;{Κ}_{T,m}$ and $E_{p,m}$, which makes us able to conclude that our data are coherent. The analysis of changes in the function $C_{p,m}=f(x_{DMF})$ with increasing concentration and temperature allows us to confirm the changes in the nature and strength of interactions between different species in the structure of the system that are occurring and which were discussed earlier. Based on these data, one can observe the transition from strong hydrogen bonds in the structure of BuOH to weak intermolecular interactions in the DMF-rich region. With the values of $\kappa_{S}$ and $\kappa_{T}$ it was also possible to calculate the values of $C_{V,m}$ according to Equation (25) [25]:

(25) $$C_{V,m}=C_{p,m}\frac{\kappa_{S}}{\kappa_{T}}$$

The obtained calculation results for $C_{V,m}$ have been collected in Table S9 in the supplementary materials.

Excess molar isobaric $\left(C_{p,m}^{E}\right)$ and isochoric $\left(C_{V,m}^{E}\right)$ heat capacities were calculated using Equations (26) and (27).

(26) $$C_{p,m}^{E}=C_{p,m}-C_{p,m}^{id}=C_{p,m}-(x_{1}\cdot C_{p,m,1}^{*}+x_{2}\cdot C_{p,m,2}^{*})$$

(27) $$C_{V,m}^{E}=C_{V,m}-C_{V,m}^{id}=C_{V,m}-C_{p,m}^{id}\cdot \frac{\kappa_{S}^{id}}{\kappa_{T}^{id}}$$

where: $C_{p,m}^{id},\;C_{V,m}^{id}$ are the isobaric and isochoric molar heat capacities of the ideal mixture, $\kappa_{T}^{id}$, $\kappa_{S}^{id}$ are the isothermal and isentropic compressibility coefficients for the ideal mixture: and $C_{p,m,i}^{*},\;C_{V,m,i}^{*}$ are the isobaric and isochoric molar heat capacities of pure compounds, BuOH (1) and DMF (2).

**Figure 7:**
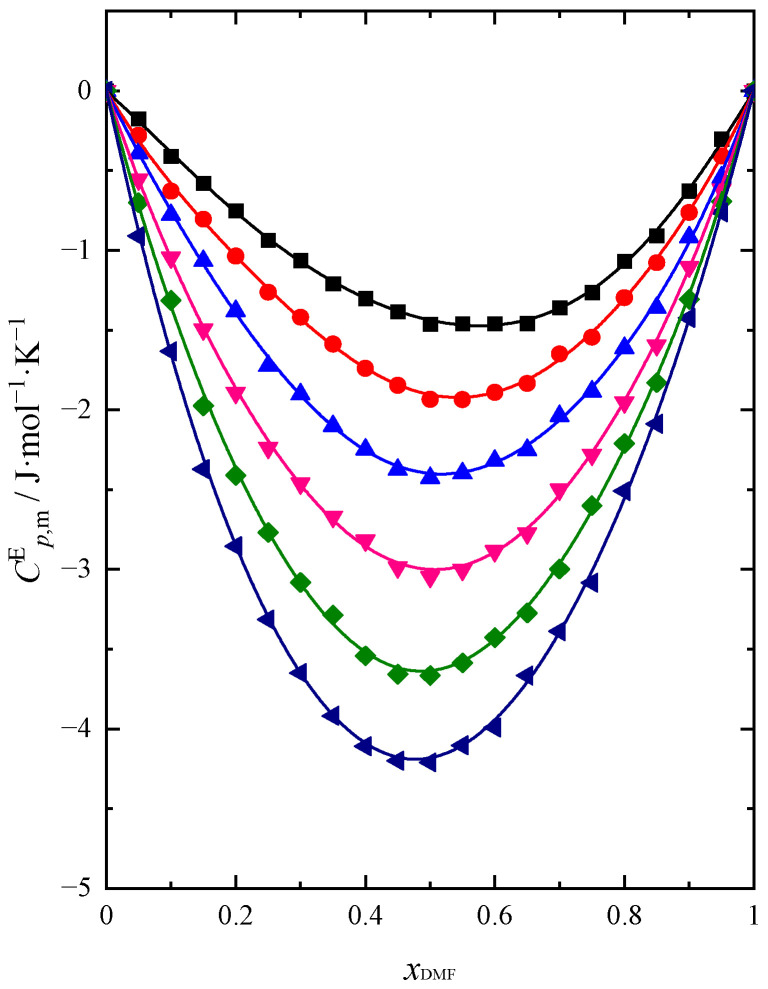
Excess molar heat capacity ($C_{p,m}^{E}$) of (DMF + BuOH) mixture: ■ *T* = 293.15 K, ● *T* = 298.15 K, ▲ *T* = 303.15 K, ▼ *T* = 308.15 K, ◆ *T* = 313.15 K, ◀ *T* = 318.15 K. Solid lines are obtained using Redlich–Kister Equation (4).

**Figure 8:**
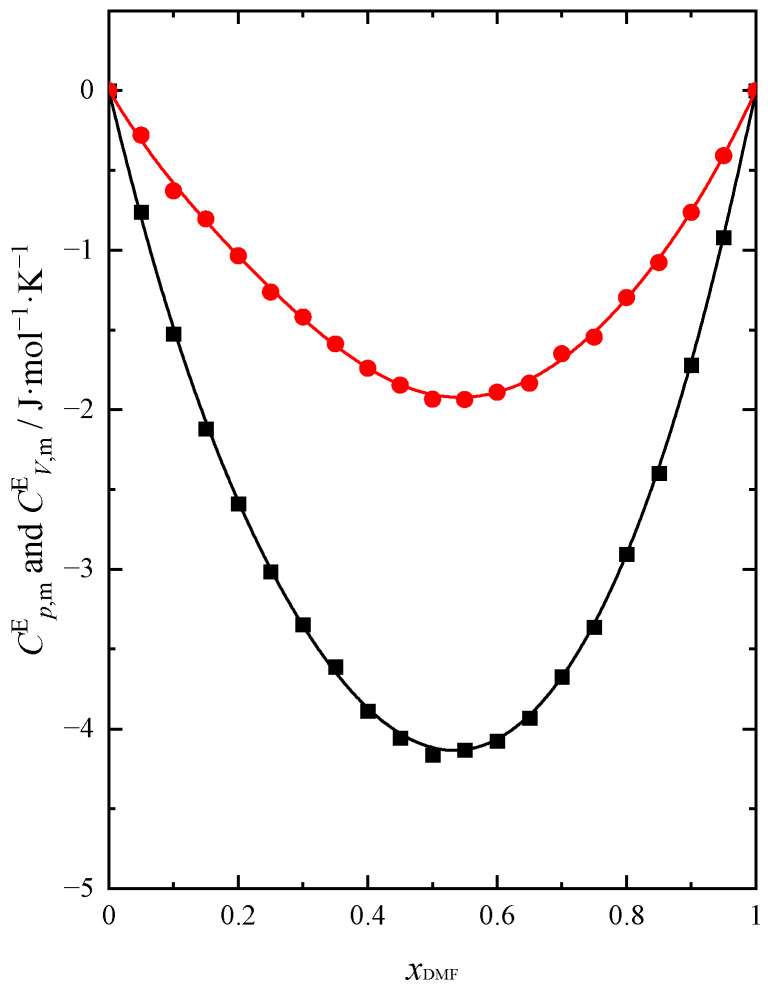
Excess molar isobaric ${(C}_{p,m}^{E}$) *T* = 293.15 K, ● and isochoric ${(C}_{V,m}^{E}$) ■ heat capacity of DMF + BuOH mixture at 298.15 K. Solid lines are obtained using Redlich-Kister Equation (4).

$C_{p,m}^{E}$ is presented as a function of mixture composition in Figure 7. The obtained dependency of $C_{V,m}^{E}=f(x_{DMF})$ is analogous to $C_{p,m}^{E}=f(x_{DMF})$ with the change in temperature. A comparison of both functions at 298.15 K is shown in Figure 8.

The excess molar isobaric ${(C}_{p,m}^{E}$) and isochoric ${(C}_{V,m}^{E}$) heat capacities of the DMF + BuOH mixture show negative values in the entire composition range of the mixture. The reason for this reduction of $C_{p,m}^{E}$ is the formation of interactions between DMF and BuOH molecules, which are presumably weaker compared to the hydrogen bonds between the molecules of these compounds in their pure form. As the temperature increases, we observe an increase in the negative values of both functions. With increasing DMF content, $C_{p,m}^{E}$ and $C_{V,m}^{E}$ decrease and reach a minimum value when $x_{DMF}$ ≈ 0.5. The obtained results confirm the changes in $K_{S,m}^{E}$, $K_{T,m}^{E}$ and $E_{p,m}^{E}$ already analyzed in this paper. All analyzed excess functions, including $C_{p,m}^{E}$ and $C_{V,m}^{E}$ reach their extreme value when $x_{DMF}$ ≈ 0.5. The obtained results of thermal properties for the DMF + BuOH mixture confirm the characteristic change in the strength of interactions between the components of the mixture with the change in the composition of the system. Interactions in the system weaken in the BuOH-rich region with an increasing amount of DMF. This allows us to confirm the conclusion that, due to the specific arrangement of neighboring molecules around the DMF and BuOH molecules forming the bond, this interaction is weakened [30]. Hence, we observe $C_{p,m}^{E}$ and $C_{V,m}^{E}$ negative values. Then one can notice an increase in the strength of the interaction when DMF is prevailing. Otherwise, as a result of the formation of stronger intermolecular hydrogen bonds between different molecules than in their pure state, the maximum would be observed [33]. In Figure 7, the position of the minimum shifts very slightly towards lower values of the DMF with increasing temperature. An analogous course is observed for $C_{V,m}^{E}=f\left(x_{DMF}\right)$ dependence.

## 3. Experimental

### 3.1. Materials

The list of compounds used in the study in this paper, as well as the suppliers, purity, purification method, and water content, is presented in Table 1.

### 3.2. Method

#### 3.2.1. Density and Speed of Sound

The density and speed of sound of the DMF + BuOH mixture within its entire concentration range were measured at temperatures *T* = (293.15, 298.15, 303.15, 308.15, 313.15, 318.15) K with the use of a DSA 5000 analyzer from Anton Paar. It is a device that allows simultaneous measurement of the density and speed of sound of a liquid sample at ambient pressure by connecting the two measuring cells in a linear manner. These cells are located in the thermostat block. The repeatability of the temperature measurement declared by the manufacturer is ±0.001 K, while its uncertainty is 0.01 K. The range of density measurement of the Anton Paar DSA 5000 M densimeter is (0–3) g·cm^−3^. The repeatability of the density measurement declared by the manufacturer is ±1 × 10^−3^ kg·m^−3^ and the determined uncertainty is equal ±2 × 10^−2^ kg·m^−3^ considering the formula for the combined standard uncertainty for the average density measurements proposed by Fortin et al. [38]. Each series of measurements was preceded by checking the correct device operation. The verification consisted of measuring the density and speed of ultrasound propagation of ultra-pure degassed water (direct Q3 UV purification). Particular attention was paid to cleaning the entire system between measurements. Due to the combination of cells, this is extremely important. To avoid the appearance of gas bubbles, which would significantly disturb the entire measurement, the density of the tested solution was measured from the highest temperature (318.15 K) to the lowest temperature (293.15 K) with increments of five degrees. The speed of sound of the DMF + BuOH mixture was also measured in the temperature range (293.15–318.15) K. The measurement uncertainty is ±0.5 m·s^−1^, and its precision is estimated to ±0.1 m·s^−1^. The speed of sound is measured by measuring the propagation time of the sound signal. The sound speed cell has a circular cylindrical cavity of 8 mm diameter and 5 mm thickness. The sample is introduced between two piezoelectric ultrasonic transducers. The first transducer produces sound waves (the average frequency is 3 MHz), while the second transducer receives the waves. If necessary, calibration of the measuring equipment was also carried out according to the procedure in accordance with the manufacturer’s instructions using ultra-pure, degassed water and air at 293.15 K and at 0.1002 MPa pressure. The values of water density and speed of sound, amounting to 998.204 kg·m^−3^ and 1482.63 m·s^−1^ at a temperature of 293.15 K, are consistent with those given in our previous paper [26].

The DMF + BuOH solutions were prepared by weight using a Sartorius Quintrix 125D-1CEU balance. The readability and repeatability of the mass measurement were, respectively, 1 × 10^−5^ g and 4 × 10^−5^ g. After the weight was determined, the sample was weighed until the same result appeared three times. The uncertainty of the determined value of the mole fraction of the component in the solution is ±5 × 10^−5^. In order to obtain a solution of a certain composition, suitable components were collected using a syringe. Solvents were degassed in an ultrasonic bath prior to use. Particular attention was paid to high-accuracy weighing and thorough cleaning of the measuring cells after each measurement to maintain this level of performance. The data on solution densities and speed of sound obtained as a function of DMF mole fraction ($x_{DMF}$) and temperature (*T*) are presented in Tables S1 and S2 (supplementary material). The mentioned data obtained by us for pure DMF and BuOH are compared with the data from the literature in Table 2.

#### 3.2.2. Heat Capacity

The measurement of the specific heat capacity, $c_{p}$, of DMF + BuOH mixtures as well as $c_{p}$ of pure solvents was carried out by means of a differential scanning calorimeter Micro DSC III manufactured by Setaram and based on Tian–Calvet’s principle. The detailed description of the measurement procedure has been described by Góralski et al. [82]. We recorded the heat flow during sample heating from 288.15 K to 323.15 K with a scanning rate of 0.35 K·min^−1^. The so-called continuous with reference method was used, with a known capacity of 1-butanol as a reference substance [83]. The uncertainty of the absolute temperature value in the measuring cell is estimated to be (0.05 K). The temperature of the external cooling system is kept constant (0.02 °K) with a HAAKE type DC30 thermostat. The measuring vessel was the standard ‘batch’ type cell with a volume ~1.0 cm^3^. The uncertainty in the $c_{p}$ values can be estimated to be smaller than 0.5%, excluding the effects of sample impurities [83] and the error of the absolute temperature determination of 0.05 K. The accuracy in the present investigation of $C_{p,m}$ value for DMF is ±0.8% and for BuOH is ±2.5% [83]. The values of the specific heat capacity as a function of the mole fraction of DMF are presented in Table S3 (supplementary materials). The calculated molar heat capacity data obtained by us for pure DMF and BuOH are compared with literature data in Table 3.

## 4. Conclusions

The presented work contains data and conclusions obtained using three different research methods. The results complement each other and allow one to confirm the conclusions drawn regarding changes occurring in the solution under the influence of adding the second component of the mixture and temperature. In addition, the obtained results are enriched with $c_{p}$ data, so far not available in the literature for the DMF + BuOH system, along with the change in composition and temperature. Based on density studies, sound velocity, and specific heat capacity of the DMF + BuOH mixture at six temperatures between 293.15 and 318.15 K, the excess functions such as $V_{m}^{E},E_{p,m}^{E},K_{S,m}^{E},K_{T,m}^{E},C_{p,m}^{E},C_{V,m}^{E}$ were calculated and analyzed in terms of changes occurring in the solution structure. The analysis of changes in $V_{m}^{E}$ as a function of the DMF mole fraction showed that characteristic changes in the structure of the system appeared in the tested mixture. The observed positive trends in $V_{m}^{E}$ values indicate that the effect due to the breaking up of self-associated structures of the components of the mixtures is dominant over the effect of H-bonding and dipole–dipole interaction between different molecules. The conclusions drawn turn out to be different from those drawn in the case of the DMF + PrOH solution. An increase in chain length by one –CH_2_– group in the alcohol molecule causes expansion of the mixture volume in the BuOH-rich region. The obtained results show how a small change in the size of the alcohol molecule can affect the strength of interactions and show the opposite tendency of the DMF + PrOH system to mutual accommodation. Additionally, the analysis of the excess partial molar volume of both components in the mixture confirms the changes in intermolecular interactions of the system, especially in the DMF-rich region. All the conclusions drawn were confirmed in the course of the remaining excess functions, making the obtained results reliable and consistent. The extrema appearing in the course of the other analyzed excess functions confirm the tendency of the system to form probably only weak hydrogen bonds between DMF and BuOH molecules and the fact that their strength changes depending on the DMF content in the mixture. Moreover, the disruption effect dominates in relation to the influence of differentiated intermolecular interactions in the studied system. Furthermore, the thermal analysis of the DMF + BuOH system ($C_{p,m},\;C_{V,m}$) provides previously unpublished data that confirms the nature of the interactions that are forming in the mixture. Despite the commonly accepted fact that the interactions in pure DMF are weak and there are no hydrogen bonds, they seem to gain importance in the studied system, especially when $x_{DMF}$ > 0.5.

## Figures and Tables

**Table 1:** Materials.

Name of Compound	Source	Purity ^a^	Purification Method	Mass Fraction of Water ^b^
1-butanol (BuOH)	Sigma-Aldrich (Poznan-Poland)	>0.998		3 × 10^−3^
*N*,*N*-dimethylformamide (DMF)	Sigma-Aldrich (Poznan-Poland)	0.998	Distillation under reduced pressure	2 × 10^−4^

^a^ Declared by the supplier. ^b^ Determined by the Karl Fischer method.

**Table 2:** Densities (*ρ*) and sound velocities (*u*) of BuOH and DMF at pressure *p* = 0.1002 ± 0.005 MPa ^a^.

	BuOH				DMF			
*T*/K	ρ (kg·m^−3^)		u (m·s^−1^)		ρ (kg·m^−3^)		u (m·s^−1^)	
	Exp.	Lit.	Exp.	Lit.	Exp.	Lit.	Exp.	Lit.
293.15	809.55	809.17 [39]	1256.4	1256 [40]	948.69	948.742 [41]	1477.0	1499 [18]
		809.5 [42]		1256 [42]		948.747 [43]		
		809.53 [44]		1256.33 [45]		948.737 [46]		
		809.54 [19]		1256.55 [47]		948.611 [48]		
		809.58 [45]		1256.57 [44]		948.546 [49]		
		809.64 [50]		1260.4 [18]		948.584 [51]		
		809.664 [47]				948.67 [19]		
		809.7 [52]				948.73 [52]		
		809.78 [15]				948.653 [53]		
		809.79 [54]						
		809.8 [40]						
		809.94 [55]						
298.15	805.74	805.54 [39]	1239.5	1239.28 [56]	943.92	943.976 [41]	1457.4	1469.5 [57]
		805.6 [16]		1239.29 [45]				
		805.73 [19]		1240.09 [58]		943.981 [43]		1457.69 [59]
		805.76 [60]		1240.2 [61]		943.971 [46]		1458.5 [62]
		805.77 [50]		1240.37 [63]		944.290 [59]		1457.49 [64]
		805.79 [45]		1240.52 [47]		943.869 [65]		1458 [66]
		805.806 [67]		1240.55 [68]		943.817 [51]		1457.13 [49]
		805.81 [63]		1241 [42]		944.603 [66]		1478 [18]
		805.851 [47]		1241.6 [18]		943.90 [19]		1465.2 [61]
		805.89 [69]		1277.55 [50]		943.97 [52]		
		805.94 [70]				944.09 [70]		
		806 [52]						
		806.06 [68]						
303.15	801.90	801.90 [39]	1222.7	1222.36 [45]	939.14	939.201 [41]	1438.0	1438.23 [59]
		801.90 [19]		1222.5 [71]		939.206 [43]		1440.2 [62]
		801.92 [71]		1223 [40]		939.196 [46]		1476.2 [72]
		801.94 [73]		1223.69 [47]		939.047 [59]		1458.8 [18]
		801.95 [45]		1224 [42]		939.073 [48]		
		801.970 [67]		1229.2 [18]		939.042 [51]		
		802.009 [47]				939.13 [19]		
		802.04 [15]				939.8 [73]		
		802.28 [55]						
		802.31 [14]						
308.15	798.03	789.03 [19]	1206.0	1206.5 [44]	934.36	934.425 [41]	1418.6	1421.95 [59]
		789.25 [39]		1206.5 [71]		934.430 [43]		1420.8 [62]
		798 [61]		1205.54 [45]		934.420 [46]		1426.03 [74]
		798.06 [44]		1206.95 [47]		934.721 [59]		1464.6 [72]
		798.10 [45]		1215.6 [18]		934.298 [65]		1432.4 [18]
		798.101 [67]				934.255 [51]		
		798.133 [47]				934.34 [19]		
		798.21 [75]				941.2 [52]		
		798.25 [39]						
		798.41 [55]						
313.15	794.13	794.12 [19]	1189.4	1188.65 [76]	929.56	929.478 [59]	1399.4	1395.69 [59]
		794.195 [67]		1188.81 [45]		929.458 [51]		1408.8 [18]
		794.22 [45]		1189.6 [77]		929.55 [19]		
		794.42 [68]		1190.48 [68]				
		794.49 [55]		1193 [78]				
		794.60 [39]		1196.0 [18]				
318.15	790.18	790.24 [45]	1173.2	1172.19 [45]	924.76	924.683 [65]	1380.4	1453.5 [72]
		790.248 [55]		1173.0 [77]		925.49 [79]		
		790.5 [61]				924.04 [80]		
		790.53 [67]				924.6 [81]		
		790.97 [39]						

^a^ Standard uncertainties *u* are *u*(*T*) = 0.01 K, *u*(*p*) = 0.005 MPa, and the combined expanded uncertainty *U*_c_ are *U*_c_(*u*) = 0.5 m·s^−1^ and *U*_c_(*ρ*) = 2 × 10^−2^ kg·m^−3^ with 0.95 level of confidence (*k* ≈ 2).

**Table 3:** Isobaric molar heat capacities ($C_{p,m}$) for BuOH and DMF at pressure *p* = 0.1001 ± 0.005 MPa.

*T*/K	*C_p,m_*/(J·mol^−1^·K^−1^)			
	BuOH		DMF	
	This Paper	Literature	This Paper	Literature
293.15	176.3	173.80 [84] 174.00 [50] 176.8 [83] ^b^ 177.6 [83] ^a^	148.3	147.16 [63] 147.3 [62] ^a^ 147.5 [62] ^b^
298.15	179.3	177.10 [84] 177.47 [50] 180.9 [83] ^b^ 181.4 [83] ^a^	148.9	148.0 [83] ^b^ 148.1 [83] ^a^ 148.16 [85] 148.2 [85] 148.54 [62] 150.16 [86]
303.15	182.7	180.61 [84] 185.2 [83] ^a^ 185.3 [83] ^b^	149.5	148.5 [83] ^b^ 148.9 [83] ^a^ 150.41 [62] 151.3 [87] 153.32 [86]
308.15	186.3	184.28 [84] 189.2 [83] ^a^ 189.8 [83] ^b^	150.2	149.1 [83] ^b^ 149.8 [83] ^a^ 152.65 [62]
313.15	190.1	188.02 [84] 193.2 [83] ^a^ 194.4 [83] ^b^	150.9	149.7 [83] ^b^ 150.7 [83] ^a^ 152.9 [87]
318.15	193.9	192.10 [84] 197.3 [83] ^a^ 199.1 [83] ^b^	151.6	150.4 [83] ^b^ 151.5 [83] ^a^

^a^ data calculated from recommended values of parameters of quasi-polynomial equation: $\frac{C_{p,m}}{R}=A_{1}\ln\left(1-T_{r}\right)+\frac{A_{2}}{1-T_{r}}+\sum_{j=0}^{m}A_{j+3}T_{r}^{j}$ where: R—gas constant, $T_{r}=\frac{T}{T_{c}}$, $T_{c}$—critical temperature. ^b^ data calculated from recommended values of parameters of cubic spline polynomials or parameters of regression polynomials: $\frac{C_{p,m}}{R}=\sum_{j=0}^{n}A_{j+1}{\left(\frac{T}{100}\right)}^{j}$.

## Data Availability

Not applicable.

## Supplementary Materials

The following supporting information can be downloaded at: https://www.mdpi.com/article/10.3390/molecules28124698/s1, Table S1. Experimental values of density, *ρ*, of (DMF + BuOH) mixtures at different temperature at pressure *p* = 0.1002 ± 0.005 MPa. Table S2. Experimental values of sound velocity, *u*, for (DMF + BuOH) mixtures at different temperature at pressure *p* = 0.1002 ± 0.005 MPa. Table S3. Experimental values of specific heat capacity, $c_{p}$, of (DMF + BuOH) mixtures at different temperature at pressure *p* = 0.1002 ± 0.005 MPa. Table S4. Partial molar volume of DMF, $V_{m,DMF}$, for (DMF + BuOH) mixtures at different temperature at pressure *p* = 0.1002 ± 0.005 MPa. Table S5. Partial molar volume of BuOH, $V_{m,BuOH},$ for (DMF + BuOH) mixtures at different temperature at pressure *p* = 0.1002 ± 0.005 MPa. Table S6. Excess partial molar volume of DMF, $V_{m,DMF}^{E}$, for (DMF + BuOH) mixtures at different temperature at pressure *p* = 0.1002 ± 0.005 MPa. Table S7. Excess partial molar volume of BuOH, $V_{m,BuOH}^{E},$ for (DMF + BuOH) mixtures at different temperature at pressure *p* = 0.1002 ± 0.005 MPa. Table S8. The values of isentropic (*κ*_s_) and isothermal (*κ*_T_) compressibility coefficients for (DMF + BuOH) mixtures at different temperature at pressure *p* = 0.1002 ± 0.005 MPa. Table S9. Isobaric (*C_p_*_,m_) and isochoric (*C_V_*_,m_) molar heat capacity of (DMF + BuOH) mixtures at different temperature at pressure *p* = 0.1002 ± 0.005 MPa.
