# Supplementary material for: Thermodynamic and Thermal Analyze of N,N-Dimethylformamide + 1-Butanol Mixture Properties Based on Density, Sound Velocity and Heat Capacity Data
Source: Molecules. 2023 Jun 11;28(12):4698. doi: 10.3390/molecules28124698 (PMC10305568; doi:10.3390/molecules28124698)
Supplement: Supplementary file 1 [file molecules-28-04698-s001.zip › molecules-2433921-supplementary.pdf]

Supplementary Material for

Thermodynamic and Thermal Analyze of *N,N*-Dimethylformamide + 1-Butanol Mixture Properties Based on Density, Sound Velocity and Heat Capacity Data

Magdalena Tyczyńska *, Aleksandra Dentkiewicz and Małgorzata Jóźwiak

Department of Physical Chemistry, Faculty of Chemistry, University of Lodz, Pomorska 165, 90-236 Lodz, Poland

* Correspondence: magdalena.tyczynska@chemia.uni.lodz.pl

Table S1. Experimental values of density, ρ, of (DMF + BuOH) mixtures at different temperature at pressure p = 0.1002 ± 0.005 MPa [a].

	ρ/ kg·m^{-3}					
x_{DMF}	293.15 K	298.15 K	303.15 K	308.15 K	313.15 K	318.15 K
0.0000	809.55	805.74	801.90	798.03	794.13	790.18
0.0500	815.47	811.59	807.69	803.76	799.79	795.79
0.1000	821.43	817.49	813.52	809.53	805.51	801.46
0.1500	827.50	823.49	819.46	815.41	811.34	807.24
0.1999	833.67	829.61	825.53	821.43	817.30	813.15
0.2499	839.94	835.84	831.71	827.55	823.38	819.19
0.3000	846.34	842.18	838.00	833.80	829.58	825.34
0.3499	852.85	848.65	844.42	840.18	835.92	831.63
0.4000	859.49	855.25	850.97	846.68	842.37	838.04
0.4500	866.25	861.96	857.64	853.30	848.94	844.57
0.5000	873.12	868.78	864.41	860.03	855.63	851.22
0.5500	880.10	875.72	871.31	866.88	862.45	857.99
0.6000	887.21	882.79	878.33	873.86	869.37	864.88
0.6500	894.45	889.98	885.47	880.96	876.43	871.89
0.7001	901.82	897.29	892.75	888.19	883.63	879.05
0.7500	909.31	904.75	900.16	895.57	890.96	886.35
0.8000	916.96	912.35	907.72	903.09	898.45	893.79
0.8500	924.73	920.07	915.41	910.74	906.05	901.36
0.8999	932.64	927.95	923.24	918.53	913.81	909.08
0.9500	940.67	935.95	931.21	926.46	921.70	916.93
1.0000	948.69	943.92	939.14	934.36	929.56	924.76

[a] Standard uncertainties u are $u(T)$ = 0.01 K, $u(p)$ = 0.005 MPa, and the combined expanded uncertainty U_c is $U_c(\rho) = 2\times10^{-2}$ kg·m^{-3} U_c (x_{DMF}) = 5×10^{-5} with 0.95 level of confidence ($k \approx$ 2).

Table S2. Experimental values of sound velocity, u, for (DMF+BuOH) mixtures at different temperature at pressure p = 0.1002 ± 0.005 MPa [a].

	u/ m·s^{-1}					
x_{DMF}	293.15 K	298.15 K	303.15 K	308.15 K	313.15 K	318.15 K
0.0000	1256.4	1239.5	1222.7	1206.0	1189.4	1173.2
0.0500	1266.9	1249.7	1232.7	1215.8	1199.1	1182.6
0.1000	1277.0	1259.7	1242.5	1225.5	1208.6	1191.9
0.1500	1287.0	1269.6	1252.2	1235.0	1218.0	1201.3
0.1999	1297.3	1279.7	1262.3	1244.9	1227.7	1210.7
0.2499	1307.2	1289.5	1271.9	1254.5	1237.1	1220.1
0.3000	1317.4	1299.6	1281.9	1264.3	1246.8	1229.6
0.3499	1327.9	1309.9	1292.0	1274.3	1256.7	1239.4
0.4000	1338.5	1320.4	1302.4	1284.5	1266.8	1249.3
0.4500	1349.4	1331.2	1313.1	1295.1	1277.1	1259.4
0.5000	1360.3	1341.8	1323.5	1305.4	1287.4	1269.5
0.5500	1370.9	1352.4	1334.0	1315.7	1297.6	1279.7
0.6000	1382.1	1363.4	1344.9	1326.5	1308.2	1290.2
0.6500	1393.4	1374.6	1356.0	1337.4	1319.0	1300.9
0.7001	1405.0	1386.0	1367.2	1348.6	1330.0	1311.7
0.7500	1416.8	1397.8	1378.8	1360.0	1341.3	1322.8
0.8000	1428.7	1409.5	1390.4	1371.5	1352.7	1334.1
0.8500	1440.7	1421.5	1402.3	1383.2	1364.2	1345.6
0.8999	1453.0	1433.6	1414.3	1395.1	1376.1	1357.3
0.9500	1465.5	1446.0	1426.7	1407.4	1388.1	1369.1

[a] Standard uncertainties u are $u(T)$ = 0.01 K, $u(p)$ = 0.005 MPa, and the combined expanded uncertainty U_c is $U_c(u)$ = 0.5 m·s^{-1}, U_c (x_{DMF}) = 5×10^{-5} with 0.95 level of confidence ($k \approx 2$).

Table S3. Experimental values of specific heat capacity, c_p, of (DMF + BuOH) mixtures at different temperature at pressure p = 0.1002 ± 0.005 MPa [a].

x_{DMF}	c_p/ J·g^{-1}·K^{-1}					
	293.15 K	298.15 K	303.15 K	308.15 K	313.15 K	318.15 K
0.0000	2.378	2.419	2.465	2.514	2.564	2.616
0.0500	2.359	2.396	2.439	2.484	2.530	2.577
0.1000	2.338	2.373	2.413	2.454	2.497	2.540
0.1500	2.319	2.352	2.388	2.425	2.464	2.503
0.1999	2.299	2.330	2.363	2.397	2.433	2.470
0.2499	2.279	2.308	2.338	2.370	2.403	2.437
0.3000	2.260	2.286	2.315	2.344	2.374	2.405
0.3499	2.241	2.265	2.291	2.318	2.347	2.374
0.4000	2.222	2.244	2.268	2.293	2.318	2.345
0.4500	2.204	2.224	2.245	2.268	2.292	2.317
0.5000	2.185	2.203	2.224	2.244	2.266	2.289
0.5500	2.168	2.184	2.203	2.222	2.242	2.264
0.6000	2.150	2.166	2.183	2.201	2.220	2.238
0.6500	2.133	2.147	2.163	2.179	2.196	2.215
0.7001	2.116	2.131	2.145	2.160	2.175	2.192
0.7500	2.100	2.113	2.126	2.140	2.155	2.168
0.8000	2.085	2.097	2.109	2.121	2.135	2.149
0.8500	2.070	2.081	2.091	2.103	2.115	2.127
0.8999	2.056	2.066	2.076	2.086	2.097	2.109
0.9500	2.043	2.051	2.060	2.070	2.080	2.090
1.0000	2.029	2.038	2.046	2.055	2.064	2.073

[a] Standard uncertainties u are $u(T)$ = 0.01 K, $u(p)$ = 0.005 MPa, and the combined expanded uncertainty U_c is $U_c(c_p)$ = 0.0025 J·g^{-1}·K^{-1}, $U_c(x_{DMF})$ = 5×10^{-5} with 0.95 level of confidence ($k \approx 2$).

Table S4. Partial molar volume of DMF, $V_{m,DMF}$, for (DMF + BuOH) mixtures at different temperature at pressure p = 0.1002 ± 0.005 MPa.

	$V_{m,DMF}$ ×10^{5}/ m^{3}·mol^{-1}					
x_{DMF}	293.15 K	298.15 K	303.15 K	308.15 K	313.15 K	318.15 K
0.0000	7.7060	7.7484	7.7919	7.8357	7.8797	7.9239
0.0500	7.7054	7.7476	7.7908	7.8343	7.8780	7.9219
0.1000	7.7055	7.7475	7.7903	7.8335	7.8770	7.9206
0.1500	7.7055	7.7473	7.7899	7.8327	7.8759	7.9193
0.1999	7.7055	7.7470	7.7891	7.8317	7.8745	7.9177
0.2499	7.7055	7.7466	7.7884	7.8306	7.8732	7.9160
0.3000	7.7053	7.7461	7.7877	7.8295	7.8718	7.9143
0.3499	7.7051	7.7456	7.7868	7.8283	7.8702	7.9125
0.4000	7.7047	7.7449	7.7858	7.8271	7.8688	7.9108
0.4500	7.7043	7.7443	7.7849	7.8259	7.8674	7.9092
0.5000	7.7040	7.7438	7.7841	7.8249	7.8662	7.9078
0.5500	7.7038	7.7433	7.7834	7.8240	7.8650	7.9065
0.6000	7.7036	7.7430	7.7829	7.8233	7.8642	7.9055
0.6500	7.7034	7.7426	7.7824	7.8227	7.8634	7.9046
0.7001	7.7032	7.7424	7.7820	7.8221	7.8627	7.9037
0.7500	7.7031	7.7421	7.7817	7.8216	7.8621	7.9030
0.8000	7.7028	7.7419	7.7813	7.8212	7.8616	7.9024
0.8500	7.7029	7.7418	7.7812	7.8210	7.8614	7.9022
0.8999	7.7029	7.7418	7.7811	7.8210	7.8613	7.9021
0.9500	7.7031	7.7420	7.7813	7.8212	7.8615	7.9023
1.0000	7.7048	7.7437	7.7831	7.8230	7.8634	7.9042

x_{DMF} – molar fraction of *N*,*N*-dimethyloformamide; The uncertainty of the mole fraction x_W is equal ±5×10^{-5}.

Table S5. Partial molar volume of BuOH, $V_{\mathrm{m,BuOH}}$, for (DMF + BuOH) mixtures at different temperature at pressure p = 0.1002 ± 0.005 MPa.

	$V_{\mathrm{m,BuOH}}$ ×10^5/ m^3·mol^{-1}					
x_{DMF}	293.15 K	298.15 K	303.15 K	308.15 K	313.15 K	318.15 K
0.0000	9.1559	9.1992	9.2432	9.2881	9.3337	9.3803
0.0500	9.1556	9.1991	9.2433	9.2882	9.3340	9.3807
0.1000	9.1560	9.1997	9.2439	9.2890	9.3349	9.3816
0.1500	9.1564	9.2001	9.2446	9.2898	9.3357	9.3825
0.1999	9.1566	9.2005	9.2450	9.2902	9.3363	9.3832
0.2499	9.1569	9.2008	9.2454	9.2907	9.3368	9.3838
0.3000	9.1571	9.2010	9.2457	9.2911	9.3373	9.3844
0.3499	9.1571	9.2012	9.2459	9.2914	9.3377	9.3849
0.4000	9.1570	9.2012	9.2461	9.2918	9.3382	9.3855
0.4500	9.1570	9.2013	9.2463	9.2922	9.3387	9.3862
0.5000	9.1570	9.2014	9.2467	9.2927	9.3394	9.3870
0.5500	9.1570	9.2016	9.2471	9.2933	9.3402	9.3880
0.6000	9.1571	9.2019	9.2477	9.2941	9.3413	9.3892
0.6500	9.1573	9.2023	9.2483	9.2950	9.3425	9.3906
0.7001	9.1573	9.2027	9.2490	9.2960	9.3436	9.3921
0.7500	9.1575	9.2032	9.2498	9.2971	9.3450	9.3936
0.8000	9.1576	9.2036	9.2506	9.2982	9.3464	9.3953
0.8500	9.1579	9.2042	9.2516	9.2995	9.3481	9.3974
0.8999	9.1582	9.2049	9.2526	9.3010	9.3499	9.3995
0.9500	9.1588	9.2058	9.2540	9.3027	9.3521	9.4020
1.0000	9.1608	9.2082	9.2569	9.3061	9.3559	9.4062

x_{DMF} – molar fraction of *N*,*N*-dimethyloformamide; The uncertainty of the mole fraction x_{W} is equal ±5×10^{-5}.

Table S6. Excess partial molar volume of DMF, $V^{E}_{m,DMF}$, for (DMF + BuOH) mixtures at different temperature at pressure p = 0.1002 ± 0.005 MPa.

	$V^{E}_{m,DMF}$ ×10^{8}/ m^{3}·mol^{-1}					
x_{DMF}	293.15 K	298.15 K	303.15 K	308.15 K	313.15 K	318.15 K
0.0000	1.1370	4.6450	8.7737	12.6707	16.3428	19.6857
0.0500	0.5812	3.8700	7.6816	11.2658	14.6509	17.7408
0.1000	0.6986	3.7386	7.2201	10.5102	13.6136	16.4067
0.1500	0.7072	3.5512	6.7843	9.7607	12.5488	15.0771
0.1999	0.6915	3.2201	6.0229	8.6864	11.1805	13.4505
0.2499	0.6481	2.8437	5.3016	7.6363	9.8176	11.8066
0.3000	0.5131	2.3909	4.5548	6.5358	8.4086	10.1278
0.3499	0.2579	1.8336	3.6395	5.2745	6.8640	8.3462
0.4000	-0.1231	1.1868	2.6776	4.0999	5.4446	6.6195
0.4500	-0.4864	0.5752	1.7744	2.9519	4.0280	5.0252
0.5000	-0.7838	0.0581	1.0103	1.9247	2.8125	3.5963
0.5500	-1.0260	-0.4136	0.3161	1.0293	1.6784	2.3048
0.6000	-1.2408	-0.7905	-0.2101	0.3156	0.8163	1.2634
0.6500	-1.4166	-1.1366	-0.7191	-0.3299	0.0707	0.3756
0.7001	-1.6586	-1.3739	-1.0926	-0.8723	-0.6838	-0.4666
0.7500	-1.7536	-1.5990	-1.4546	-1.3508	-1.2578	-1.1572
0.8000	-1.9895	-1.8652	-1.8215	-1.7919	-1.7821	-1.7518
0.8500	-1.9570	-1.9097	-1.9187	-1.9450	-1.9665	-1.9852
0.8999	-1.9639	-1.9454	-1.9876	-2.0355	-2.0835	-2.1141
0.9500	-1.6920	-1.7271	-1.7791	-1.8286	-1.8598	-1.8978
1.0000	0.0000	0.0000	0.0000	0.0000	0.0000	0.0000

x_{DMF} – molar fraction of *N*,*N*-dimethyloformamide; The uncertainty of the mole fraction x_W is equal ±5×10^{-5}.

Table S7. Excess partial molar volume of BuOH, $V_{\mathrm{m,BuOH}}^{\mathrm{E}}$, for (DMF + BuOH) mixtures at different temperature at pressure p = 0.1002 ± 0.005 MPa.

x_{DMF}	$V_{\mathrm{m,BuOH}}^{\mathrm{E}}$ ×10^8/ m^3·mol^-1					
	293.15 K	298.15 K	303.15 K	308.15 K	313.15 K	318.15 K
0.0000	0.0000	0.0000	0.0000	0.0000	0.0000	0.0000
0.0500	-0.2535	-0.0940	0.0279	0.1291	0.2320	0.3330
0.1000	0.1661	0.4555	0.6864	0.9076	1.1186	1.2767
0.1500	0.4769	0.9490	1.3707	1.6921	1.9778	2.2249
0.1999	0.7634	1.2988	1.7294	2.1519	2.5334	2.8761
0.2499	1.0222	1.6034	2.1280	2.6358	3.0945	3.5100
0.3000	1.1894	1.8316	2.5013	3.0694	3.6094	4.1091
0.3499	1.2365	1.9551	2.7061	3.3421	3.9887	4.6053
0.4000	1.1578	1.9893	2.8641	3.7015	4.4933	5.1564
0.4500	1.0966	2.0586	3.0810	4.0875	5.0006	5.8400
0.5000	1.1015	2.2224	3.4370	4.5944	5.7091	6.6889
0.5500	1.1615	2.4317	3.8628	5.2331	6.4989	7.6752
0.6000	1.2489	2.7358	4.4566	6.0533	7.5608	8.9117
0.6500	1.3754	3.0706	5.0676	6.9419	8.7391	10.3017
0.7001	1.4356	3.5142	5.8142	7.9336	9.9085	11.7373
0.7500	1.6429	3.9701	6.5722	8.9891	11.2584	13.3245
0.8000	1.7091	4.3848	7.3254	10.0820	12.6581	15.0077
0.8500	2.0438	5.0212	8.3482	11.4629	14.3976	17.0522
0.8999	2.3392	5.6664	9.3993	12.9065	16.2046	19.2011
0.9500	2.9133	6.5657	10.7278	14.6474	18.3522	21.6952
1.0000	4.9075	8.9737	13.6270	18.0100	22.1360	25.8708

x_{DMF} – molar fraction of *N*,*N*-dimethyloformamide; The uncertainty of the mole fraction x_{W} is equal ±5×10^{-5}.

Table S8. The values of isentropic (κ_S) and isothermal (κ_T) compressibility coefficients for (DMF + BuOH) mixtures at different temperature at pressure p = 0.1002 ± 0.005 MPa.

x_{DMF}	(Pa^{-1})											
	293.15 K		298.15 K		303.15 K		308.15 K		313.15 K		318.15 K	
	$\kappa_S\times10^{10}$	$\kappa_T\times10^{10}$	$\kappa_S\times10^{10}$	$\kappa_T\times10^{10}$	$\kappa_S\times10^{10}$	$\kappa_T\times10^{10}$	$\kappa_S\times10^{10}$	$\kappa_T\times10^{10}$	$\kappa_S\times10^{10}$	$\kappa_T\times10^{10}$	$\kappa_S\times10^{10}$	$\kappa_T\times10^{10}$
0.0000	7.8249	9.1582	8.0784	9.4562	8.3417	9.7617	8.6156	10.0762	8.9007	10.0762	9.1945	10.7347
0.0500	7.6408	9.0043	7.8895	9.2977	8.1479	9.5986	8.4167	9.9087	8.6966	9.9087	8.9856	10.5589
0.1000	7.4648	8.8565	7.7085	9.1452	7.9620	9.4410	8.2255	9.7462	8.4996	9.7462	8.7828	10.3857
0.1500	7.2957	8.7174	7.5342	8.9993	7.7824	9.2889	8.0405	9.5883	8.3088	9.5883	8.5847	10.2136
0.1999	7.1271	8.5649	7.3601	8.8432	7.6026	9.1295	7.8550	9.4257	8.1178	9.4257	8.3903	10.0466
0.2499	6.9669	8.4213	7.1948	8.6954	7.4320	8.9782	7.6787	9.2695	7.9354	9.2695	8.2009	9.8801
0.3000	6.8076	8.2775	7.0303	8.5469	7.2622	8.8248	7.5033	9.1113	7.7542	9.1113	8.0138	9.7121
0.3499	6.6500	8.1302	6.8676	8.3960	7.0942	8.6704	7.3295	8.9528	7.5748	8.9528	7.8284	9.5460
0.4000	6.4943	7.9884	6.7067	8.2496	6.9279	8.5188	7.1579	8.7962	7.3978	8.7962	7.6459	9.3797
0.4500	6.3397	7.8434	6.5469	8.1007	6.7629	8.3661	6.9875	8.6399	7.2219	8.6399	7.4654	9.2154
0.5000	6.1900	7.7032	6.3933	7.9573	6.6045	8.2184	6.8235	8.4877	7.0521	8.4877	7.2896	9.0531
0.5500	6.0462	7.5661	6.2437	7.8154	6.4495	8.0718	6.6639	8.3374	6.8865	8.3374	7.1168	8.8915
0.6000	5.9009	7.4306	6.0938	7.6747	6.2946	7.9260	6.5037	8.1861	6.7212	8.1861	6.9462	8.7305
0.6500	5.7582	7.2933	5.9465	7.5336	6.1424	7.7813	6.3462	8.0374	6.5585	8.0374	6.7776	8.5719
0.7001	5.6175	7.1622	5.8012	7.3951	5.9922	7.6361	6.1909	7.8848	6.3976	7.8848	6.6116	8.4053
0.7500	5.4784	7.0240	5.6573	7.2530	5.8435	7.4897	6.0372	7.7341	6.2390	7.7341	6.4476	8.2464
0.8000	5.3428	6.8909	5.5169	7.1146	5.6983	7.3464	5.8869	7.5862	6.0830	7.5862	6.2858	8.0851
0.8500	5.2097	6.7574	5.3792	6.9769	5.5556	7.2048	5.7391	7.4383	5.9303	7.4383	6.1277	7.9292
0.8999	5.0789	6.6230	5.2436	6.8382	5.4150	7.0607	5.5936	7.2906	5.7793	7.2906	5.9714	7.7715
0.9500	4.9498	6.4868	5.1097	6.6984	5.2761	6.9175	5.4494	7.1427	5.6308	7.1427	5.8184	7.6170
1.0000	4.8322	6.3655	4.9878	6.5721	5.1497	6.7858	5.3182	7.0063	5.4938	7.0063	5.6749	7.4684

x_{DMF} – molar fraction of *N*,*N*-dimethyloformamide; The uncertainty of the mole fraction x_W is equal ±5×10^{-5}.

Table S9. Isobaric ($C_{p,\mathrm{m}}$) and isochoric ($C_{V,\mathrm{m}}$) molar heat capacity of (DMF + BuOH) mixtures at different temperature at pressure p = 0.1002 ± 0.005 MPa.

x_{DMF}	(J·mol^{-1}·K^{-1})											
	293.15 K		298.15 K		303.15 K		308.15 K		313.15 K		318.15 K	
	$C_{p,\mathrm{m}}$	$C_{V,\mathrm{m}}$	$C_{p,\mathrm{m}}$	$C_{V,\mathrm{m}}$	$C_{p,\mathrm{m}}$	$C_{V,\mathrm{m}}$	$C_{p,\mathrm{m}}$	$C_{V,\mathrm{m}}$	$C_{p,\mathrm{m}}$	$C_{V,\mathrm{m}}$	$C_{p,\mathrm{m}}$	$C_{V,\mathrm{m}}$
0.0000	176.27	150.61	179.30	153.17	182.68	156.11	186.32	159.31	190.08	162.66	193.88	166.06
0.0500	174.70	148.24	177.50	150.62	180.64	153.33	183.96	156.26	187.42	159.35	190.85	162.41
0.1000	173.06	145.87	175.63	148.04	178.59	150.62	181.66	153.32	184.85	156.16	188.01	158.99
0.1500	171.50	143.53	173.94	145.62	176.65	148.00	179.40	150.44	182.23	152.98	185.16	155.63
0.1999	169.93	141.40	172.19	143.31	174.68	145.46	177.20	147.67	179.84	150.02	182.56	152.46
0.2499	168.35	139.27	170.45	141.03	172.68	142.94	175.05	145.01	177.52	147.19	179.98	149.39
0.3000	166.82	137.20	168.77	138.82	170.84	140.59	173.02	142.48	175.25	144.45	177.53	146.49
0.3499	165.28	135.19	167.09	136.67	168.99	138.27	171.01	140.00	173.08	141.81	175.15	143.63
0.4000	163.79	133.16	165.42	134.48	167.18	135.96	169.05	137.57	170.87	139.14	172.84	140.89
0.4500	162.31	131.19	163.79	132.38	165.40	133.70	167.08	135.12	168.79	136.61	170.63	138.23
0.5000	160.83	129.24	162.19	130.31	163.69	131.54	165.21	132.82	166.83	134.21	168.51	135.68
0.5500	159.44	127.41	160.67	128.36	162.06	129.49	163.45	130.64	164.95	131.92	166.50	133.26
0.6000	158.04	125.51	159.20	126.40	160.48	127.45	161.76	128.52	163.15	129.70	164.49	130.87
0.6500	156.65	123.68	157.73	124.50	158.89	125.43	160.07	126.39	161.34	127.47	162.70	128.65
0.7001	155.35	121.85	156.40	122.69	157.45	123.56	158.54	124.48	159.66	125.46	160.86	126.53
0.7500	154.05	120.15	154.99	120.89	155.95	121.67	156.95	122.51	158.10	123.52	159.05	124.36
0.8000	152.85	118.51	153.72	119.20	154.56	119.89	155.47	120.66	156.53	121.59	157.51	122.47
0.8500	151.61	116.90	152.42	117.53	153.16	118.11	154.02	118.84	154.95	119.65	155.82	120.42
0.8999	150.49	115.41	151.22	115.95	151.94	116.53	152.71	117.16	153.51	117.85	154.36	118.61
0.9500	149.42	114.02	150.05	114.46	150.66	114.91	151.39	115.50	152.16	116.16	152.91	116.81
1.0000	148.33	112.60	148.94	113.04	149.55	113.49	150.20	114.01	150.90	114.60	151.56	115.16

x_{DMF} – molar fraction of *N*,*N*-dimethyloformamide; The uncertainty of the mole fraction x_{W} is equal ±5×10^{-5}.
